# Wing Morphometrics of *Aedes* Mosquitoes from North-Eastern France

**DOI:** 10.3390/insects12040341

**Published:** 2021-04-12

**Authors:** Jean-Philippe Martinet, Hubert Ferté, Pacôme Sientzoff, Eva Krupa, Bruno Mathieu, Jérôme Depaquit

**Affiliations:** 1 Faculté de Pharmacie, Université de Reims Champagne-Ardenne, SFR Cap Santé, EA7510 ESCAPE–USC VECPAR, 51 rue Cognacq-Jay, 51096 Reims, France; j.martinet.p@gmail.com (J.-P.M.); hubert.ferte@univ-reims.fr (H.F.); pacome.sientzoff@gmail.com (P.S.); 2 Arbovirus et Insectes Vecteurs, Département de Virologie, Institut Pasteur, 25–27 rue du docteur Roux, 75015 Paris, France; 3 Laboratoire de Parasitologie, Hôpital Maison-Blanche, CHU de Reims, 45 rue Cognacq-Jay, 51100 Reims, France; 4 Institut de Parasitologie et de Pathologie Tropicale de Strasbourg, DIHP UR 7292, Université de Strasbourg, 3 rue Koeberlé, 67000 Strasbourg, France; e.krupa@unistra.fr (E.K.); bmathieu@unistra.fr (B.M.)

**Keywords:** geometric morphometrics, mosquito wings, Culicidae

## Abstract

**Simple Summary:**

Mosquitoes act as vectors of arboviruses and their correct identification is very important to understanding the diseases they transmit. To date, this identification is based on several techniques that are either expensive or time consuming. Wing geometric morphometrics allow fast and accurate mosquito identification. By analyzing the pattern of wing venation, it is possible to separate mosquito species. We applied this technique on six *Aedes* mosquito species from north-eastern France. Our results show a very good differentiation of these species. The use of wing geometric morphometrics could increase the efficiency of field entomologists in case of viral outbreaks. Integrated with existing morphological identification software, it might help relocate mosquito identification from the lab to the field.

**Abstract:**

Background: In the context of the increasing circulation of arboviruses, a simple, fast and reliable identification method for mosquitoes is needed. Geometric morphometrics have proven useful for mosquito classification and have been used around the world on known vectors such as *Aedes albopictus*. Morphometrics applied on French indigenous mosquitoes would prove useful in the case of autochthonous outbreaks of arboviral diseases. Methods: We applied geometric morphometric analysis on six indigenous and invasive species of the *Aedes* genus in order to evaluate its efficiency for mosquito classification. Results: Six species of *Aedes* mosquitoes (*Ae. albopictus*, *Ae. cantans*, *Ae. cinereus*, *Ae. sticticus*, *Ae. japonicus* and *Ae. rusticus*) were successfully differentiated with Canonical Variate Analysis of the Procrustes dataset of superimposed coordinates of 18 wing landmarks. Conclusions: Geometric morphometrics are effective tools for the rapid, inexpensive and reliable classification of at least six species of the *Aedes* genus in France.

## 1. Introduction

Identification of mosquitoes is a matter of public health. Numerous mosquitoes are proven vectors of human or zoonotic arboviruses, such as dengue (DENV), chikungunya (CHIKV), West Nile (WNV) or Usutu (USUV). Recently, Southern Europe suffered autochthonous dengue epidemics [1]. These highlight the need for rapid vector identification, surveillance and control. Morphological methods, initially used for the description of original species and their comparisons, are the main means to quickly identify mosquitoes. They rely upon dichotomic/polytomous keys, illustrated simplified keys and interactive keys [2]. The latter, with regard to the European fauna, were firstly developed in 2000 [3] and were recently updated using the Xper2 software [4], leading to MosKeyTool [5]. This interactive identification key for mosquitoes of the Mediterranean region requires updates on fauna composition and morphological data, but also well-preserved specimens analyzed by expert personnel. While such morphological tools are very helpful, their routine use can turn out to be time-consuming. With the advent of molecular biology, molecular tools were developed in order to accurately identify mosquito species. Mostly based on barcoding techniques (analysis of the cytochrome oxidase I gene) [6], the sequencing and comparison of sequences with online databases (GenBank, BOLD) provide a reliable identification method [7,8]. However, some cryptic species like those of the *Culex pipiens* complex require further analysis of the ACE2 (acetylcholinesterase) gene and microsatellites to achieve accurate identification [9,10]. In addition to barcoding techniques, more precise molecular tools were developed in order to identify mosquitoes belonging to the same species complex. For instance, the multiplex allele-specific PCR technique was used to diagnose similar *Aedes* mosquitoes from the *Stegomyia* subgenus [11] and mosquitoes from the *Anopheles gambiae* and *Anopheles barbirostris* complexes [12,13]. In another area of molecular biology, loop-mediated isothermal amplification (LAMP) assays were created with possible outcomes in field surveillance of invasive species [14]. Finally, proteomic approaches have recently flourished in entomological identification. The MALDI-ToF technique has been successfully applied for mosquito (both adults and larvae) and blood-meal identification [15,16,17]. These approaches appear to be accurate, but are time-consuming, somewhat expensive and need consequent laboratory equipment to be performed. Barcoding can, however, be of help to identify collections or damaged specimens.

In the 2000s, the emergence of geometric morphometrics (GM) opened a new field in mosquito identification and analysis. GM is defined as the statistical analysis of form based on Cartesian landmark coordinates [18]. This approach is based on the analysis of point coordinates on the wings. A mathematical transformation can be used to extract data and then classify mosquito species [19]. GM became widely used after the “revolution in morphometrics” that occurred in the 1990s [20]. This technique shows a broad range of applications in biology in fields such as medical imaging, anthropology or even botany [21,22,23]. In the field of medical entomology, the use of GM made it possible to further analyze insect populations. As the emergence of arboviruses is on the rise, populations of vectors have been of interest for GM studies. Quite naturally, insect families such as Muscidae, Reduvidae, Ceratopogonidae or Culicidae have been exhaustively studied [24].

Currently, GM is used in mosquito classification and the survey of the effects of biotic and abiotic factors on mosquito populations [25,26,27,28]. However, this technique is mostly applied to the three main arbovirus vectors: *Aedes*, *Anopheles* and *Culex* mosquitoes. GM has proven reliable in the identification of the genus *Aedes*, such as *Ae. aegypti* and *Ae. albopictus* (the main vectors of dengue fever), and to compare the life and trait variations among these populations [28,29]. For the *Anopheles* genus, GM was able to improve reliable diagnosis for some sympatric *Anopheles* species in South America, for instance, *An. cruzii*, *An. homunculus* and *An. bellator* [30]. Within the *Culex* genera, reliable morphological discrimination between *Cx. pipiens* and *Cx. torrentium* relies on GM to separate females and observe the genitalia of males [31]. Since vector groups are substantially found in the GM literature, entomologists began to show interest in species of lesser epidemiological importance [32]. Nevertheless, as there is a non-negligible possibility of vector competence of these species, such studies increase preparedness in the case of unexpected arboviruses emergence. GM studies performed on vectors in metropolitan France have been mostly applied to the Psychodidae and Ceratopogonidae families, such as the genus *Phlebotomus* or *Culicoides* [33,34]. Mosquito vectors of metropolitan France belong to the genera *Aedes* and *Culex*. French *Ae. albopictus* has been assessed as an effective vector of DENV, CHIKV and ZIKV [35,36,37]. *Cx. modestus* and *Cx. pipiens* from southern France have been characterized as competent for WNV transmission [38]. However, to the best of our knowledge, none of the autochthonous or invasive populations of French *Aedes* mosquitoes have been submitted to GM analysis.

In the present study, we propose an analysis of wing traits and the classification of mosquito species endemic to north-eastern France. Our sampling challenges several arbovirus vectors (*Ae. albopictus*, *Ae. cinereus* s.l., *Ae. sticticus* and *Ae. japonicus*) [39] and includes a couple of species without any proven vector status (*Ae. cantans* and *Ae. rusticus*).

## 2. Materials and Methods

**Figure 1:**
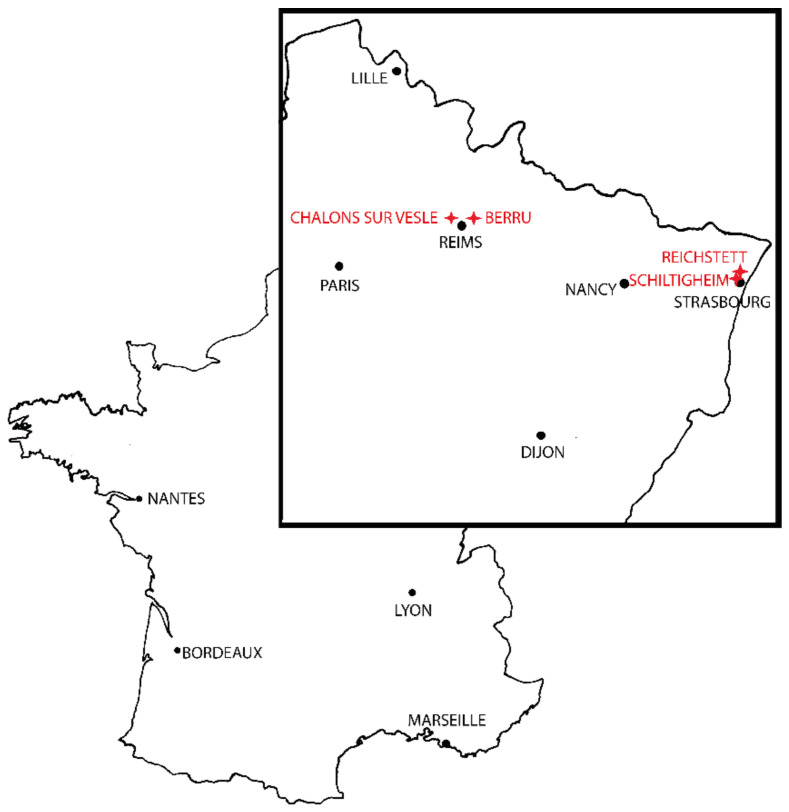
Sampling map.

Female mosquitoes were captured from 2018 to 2019 in the Grand-Est region, in the localities of Berru, Châlons-sur-Vesle, Reichstett and Schiltigheim (Figure 1). Females were collected with BG Sentinel^©^ (Biogents, Regensburg, Germany) traps and by human-landing techniques (Table 1). Samples were brought back to the laboratory and placed into cages prior to identification, except for *Ae. albopictus* and *Ae. japonicus*, which were stored in 70% ethanol until dissection and analysis. Mosquitoes were anesthetized by cold, morphologically identified at the species level using taxonomic keys (Schaffner et al. and Möhrig [3,40]) and euthanatized. Right wings were dissected under a stereomicroscope, underwent mechanical treatment to remove scales [41], dehydrated in successive ethanol baths and mounted on slides with Euparal mounting medium ^©^) (Carl Roth, Karlsruhe, Germany).

Legs were used for molecular identification. Samples were randomly chosen within each group and went through a molecular barcoding identification. DNA was extracted with the DNeasy Blood and Tissue extraction kit (Qiagen, Hilden Germany) following the manufacturer’s instruction. Polymerase Chain Reaction performed on a 648 bp fragment of the COI gene was set as follows: initial denaturation at 94 °C for 30 s, followed by 5 cycles at 94 °C for 30 s, 45 °C for 30 s and 72 °C for 1 min, then 35 cycles at 94 °C for 30 s, 51 °C for 30 s, 72 °C for 1 min and a final elongation step at 72 °C for 10 min.

The following primers were used: LEPF1 (5′-TTTCTACAAATCATAAAGATATTGG-3′) and LEPR1 (5′-TAAACTTCTGGATGTCCAAAAAATCA-3′) [42].

Amplicons went through Sanger sequencing (Genewiz, Leipzig, Germany). Sequences were compared to existing GenBanK sequences with the BLAST algorithm [43] and identification was considered accurate above a 99% similarity.

**Figure 2:**
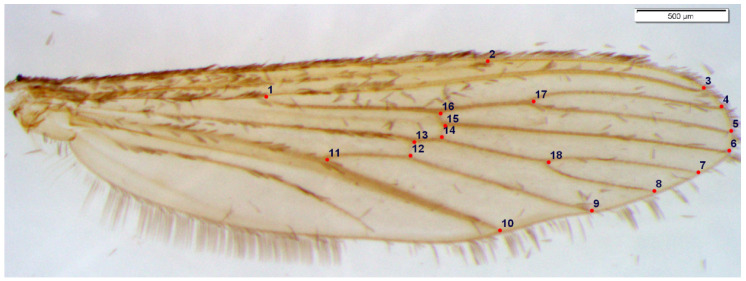
Position of the 18 landmarks (recorded from the 1st to the 18th respectively) on an *Aedes cinereus* wing (scale bar = 500 µm).

Pictures were taken using the Stream Essentials software version 1.7 and a DP-26 video camera connected to a SZX10 stereomicroscope (Olympus, Tokyo, Japan). All specimens were captured with a X2 magnification. Pictures were saved in JPEG format, and the work files were built with TPS Util^©^ version 1.76. In total, 18 landmarks were manually digitized by one of the authors (JPM) with TPSDig^©^ version 2.31 [44], as shown in Figure 2.

Error assessment: In order to evaluate the error in landmark digitization, we performed a Pearson correlation test on a subset of 76 randomly chosen pictures digitized twice by the same operator (JPM).

**Figure 3:**
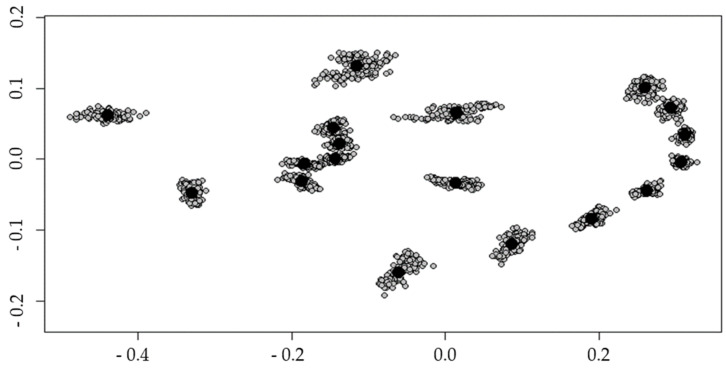
Landmark positions after Procrustes superimposition.

**Figure 4:**
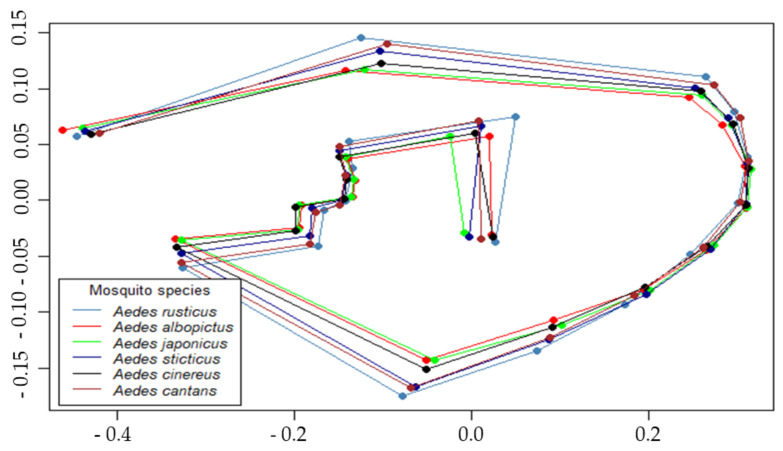
Mean position of the 18 landmarks by mosquito species.

Landmark analysis: Coordinates of the 18 landmarks were imported in RStudio software (version 1.2.5019) [45] and processed within the *geomorph* package (version 3.2.1) [46]. Coordinates were aligned by performing Procrustes superimposition (Figure 3). The mean positions of the landmarks per species are shown in Figure 4. Plots exported from R were made with the generic plot function.

Coordinates in TPS format were imported in MorphoJ software version 1.07a [47]. Multivariate regression over the Procrustes coordinates was performed in order to evaluate the allometric influence of size over shape. Canonical Variate Analysis (CVA) was applied on the coordinates and Mahalanobis distances were computed to study the similarity between species. Pairwise cross-validated species reclassification tests with 1000 permutation runs were conducted. This test aims to quantify the rate of correct reclassification between samples.

Cross-validation over Mahalanobis distances was performed, and a neighbor-joining tree, including a population of *Culex torrentium* (*n* = 14) as outgroup, was computed over 100 bootstraps using PAST v2.17c [48].

## 3. Results

### 3.1. Mosquito Collection and Identification

Taking into account their wing integrity, a total of 148 females has been selected (Table 1). Sequences of the specimens sequenced in the present study are available in Gen-Bank under accession numbers MW843020 to MW843031.

### 3.2. Error Measurement

The Pearson correlation test on our data subset showed a good repeatability of our digitization process (correlation coefficient of 0.9999639, 95 percent confidence interval: 0.9999611–0.9999665, *p*-value < 0.0001).

### 3.3. Mean Shapes

Procrustes superimposition performed on the raw coordinates made it possible to align all landmarks positions (Figure 3). For each species, the median position of each landmark was processed and allowed to draw the following composite and observe the maximum deviation for landmarks 10 to 18. (Figure 4).

### 3.4. Allometric Regression

Multivariate regression of the Procrustes coordinates on CS shows an allometric effect of wing size on wing shape (3.95%, *p* < 0.0001). We did not choose to remove it as we consider, like Wilke et al., that allometric size variation is a part of the process of species identification [19].

### 3.5. Canonical Variate Analysis

**Figure 5:**
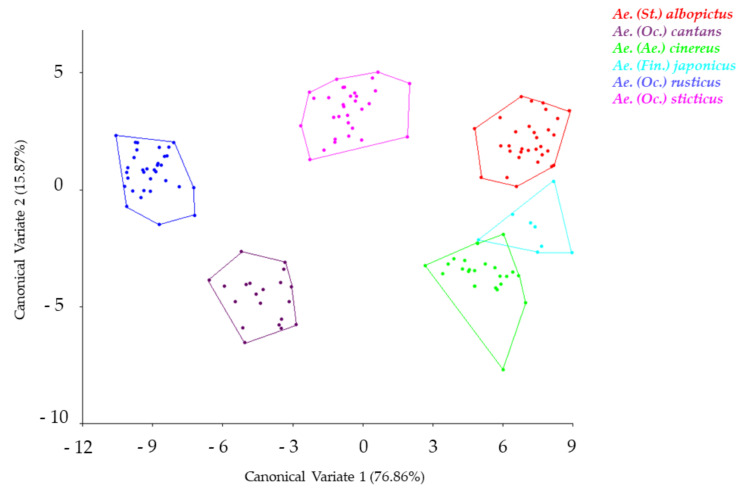
Canonical Variate Analysis of the Procrustes coordinates of *Aedes* mosquitoes.

**Figure 6:**
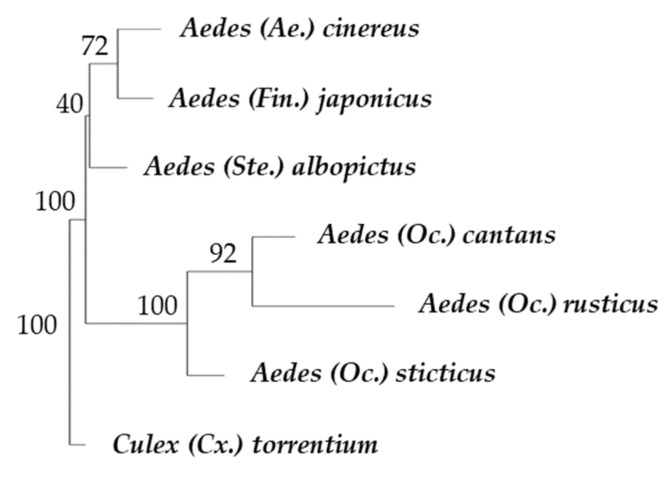
Neighbor-joining tree performed over Mahalanobis distances and computed over 100 bootstrap replicates.

Canonical Variate Analysis performed on our dataset accounted for 86.73% of the total variance on the first two canonical variates. The specimens from the six species studied here belong to four subgenera: *Ae. albopictus* belongs to the subgenus *Stegomyia*, *Ae. japonicus* to the subgenus *Finlaya*, *Ae. cinereus* s.l. to the subgenus *Aedes*, *Ae. cantans, Ae. rusticus* and *Ae. sticticus* to the subgenus *Ochlerotatus*. Figure 5 shows a relative clustering between the *Stegomyia* and *Aedes* subgenera. Species appear to be well segregated with low overlapping. The pairwise cross-validated species reclassification test shows an accuracy of 98%. The detailed pairwise cross-validated species reclassification test is available in Table 2. A neighbor-joining tree was performed on Mahalanobis distances between these species (Figure 6).

This tree shows the branching of *Ae. cantans*, *Ae. rusticus* and *Ae. sticticus*, all members of the subgenus *Ochlerotatus*, well supported by a bootstrap rate of 100%. The branch including *Ae. albopictus*, *Ae. cinereus* and *Ae. japonicus* is not supported by bootstrap. 

## 4. Discussion

In the present paper, we show that morphometric tools are efficient to classify *Aedes* mosquitoes from north-eastern France. We focused our sampling on this genus because it includes most of the vectors of mosquito-borne arboviruses. *Ae. albopictus* is an efficient vector of DENV, although less efficient than *Ae. aegypti* [49]. French populations of *Ae. albopictus* are competent for DENV [37] and can also transmit CHIKV and ZIKV [35,36]. In Germany, the Netherlands and Switzerland, *Ae. japonicus* was shown to be an effective vector of CHIKV, DENV, USUV and ZIKV [50,51,52,53]. The vector competence of *Ae. cantans*, *Ae. cinereus*, *Ae. rusticus* and *Ae. sticticus* remains mostly unknown, although *Ae. cantans* has been found positive for WNV in some recent screenings [54]. Despite the lack of data about their vector competence, these species could be locally abundant and responsible for nuisance (personal observation).

The goal of the neighbor-joining tree built (Figure 6) is not to analyze the evolution patterns of these species, as both the sampling and the methods used are not appropriate for this purpose. The tree emphasizes that the three members of the subgenus *Ochlerotatus* (*Ae. cantans*, *Ae. rusticus* and *Ae. sticticus*) are clustered together. This means that their wings share more similarities than with the wings of other species. The origin of these similarities could be of phylogenetical inheritance providing similar structures (they belong to the same subgenus) or could be linked to their wing sizes, which are the largest across our samples (personal observation). Conversely, *Ae. albopictus* and *Ae. japonicus* are branched together, despite the fact that they belong to different subgenera.

Morphometrics have been successfully used in different applications, such as the discrimination and identification of mosquitoes (including sibling species, such as *Cx. pipiens* and *Cx. torrentium* [31], or sympatric *Anopheles* [30]) and to assess the influence of biotic or abiotic factors on mosquito wings [26].

GM have proven effective in the entomological field for species differentiation or the analysis of cryptic complexes. In this study, we successfully applied geometric morphometrics on French indigenous and invasive *Aedes* wings. This technique allowed a rapid and effective classification of six species of the *Aedes* genus: *Ae. albopictus*, *Ae. cantans*, *Ae. cinereus* s.l., *Ae. japonicus*, *Ae. rusticus* and *Ae. sticticus*. GM has already been used in Europe to identify female mosquitoes of autochthonous and invasive species [55]. Nevertheless, this technique is still struggling to differentiate between closely related species, such as *Ae. annulipes* and *Ae. cantans* [19,55]. Our results are in accordance with other studies performed in Europe. 

Due to all the morphometric literature, researchers are steadily building a database of wing patterns. It would be interesting if all this worldwide data could be merged in order to create a global catalog of mosquito wing patterns. As some authors have shown, the landmark disposition of two geographically isolated mosquito populations from the same species can show pattern variation [28]. Nonetheless, such large databases could be of help to create worldwide tools for mosquito identification.

GM is a valuable tool to prepare for the emergence of arboviruses. Exhaustive databases could be built and made available to that end. Integration of GM tools into identification software (such as MosKeyTool) could help ease the process of identification, allowing beginner field entomologists to make accurate identifications, and confirmed entomologists to save valuable time in the case of an epidemic event.

## 5. Conclusions

Geometric morphometrics are a proven efficient tool in mosquito classification [19]. They allow the rapid and reliable identification of mosquito species, including closely related species and genera. Six autochthonous and invasive *Aedes* species from the north-east of France were successfully segregated in this study, with a correct reclassification rate of 98%.

As the number of morphological experts decreases, morphometric identification could be of assistance when molecular identification cannot be performed (i.e., specimens deposited in curated collections, especially type-specimens stored in museums). Today, we are witnessing an increasing number of outbreaks of mosquito-borne emerging and re-emerging diseases. In this context, field studies are mandatory to assess the presence of known vectors. Morphometrics could reduce the processing time of samples caught in the field and directly decrease latency between entomological investigation and targeted vector control operations.

Geometric morphometrics are a developing field of biological studies. The principal flaw of this technique is that landmarks must be placed manually, meaning human error is a variable in the rigorous mathematical treatment of this method. Advances in machine learning and computer vision will hopefully make it possible to automatize the entire analysis process in the near future.

## Figures and Tables

**Table 1:** Locations of the mosquito species.

Species	Collection Date	City	Latitude	Longitude	Number of Specimens
*Aedes albopictus*	19 September 2019	Shiltigheim	48.603253	7.734191	31
*Aedes cantans*	24 April 2018	Châlons/Vesle	49.288187	3.924016	20
*Aedes cinereus*	29 June 2018	Berru	49.267750	4.133623	25
*Aedes sticticus*	29 June 2018	Berru	49.267750	4.133623	31
*Aedes japonicus*	1 October 2019	Reichstett	48.648827	7.757608	8
*Aedes rusticus*	23 May 2018	Berru	49.267750	4.133623	33

Sequences of the specimens sequenced in the present study are available in GenBank under accession numbers MW843020 to MW843031.

**Table 2:** Pairwise cross-validated species reclassification test. Values below the diagonal correspond to the proportion of Group 1 specimens correctly identified after comparison with Group 2. Values above the diagonal correspond to the proportion of Group 2 specimens correctly identified after comparison with Group 1.

Reclassification Test		Group 2					
		*Aedes albopictus*	*Aedes cantans*	*Aedes cinereus*	*Aedes sticticus*	*Aedes japonicus*	*Aedes rusticus*
**Group 1**	*Aedes albopictus*	×	100%	100%	100%	75%	100%
	*Aedes cantans*	97%	×	100%	94%	100%	100%
	*Aedes cinereus*	100%	95%	×	100%	100%	100%
	*Aedes sticticus*	97%	100%	100%	×	100%	100%
	*Aedes japonicus*	90%	100%	96%	100%	×	100%
	*Aedes rusticus*	100%	100%	100%	97%	100%	×

The pairwise cross-validated reclassification test was efficient to separate the specimens. The lowest values were obtained between *Ae. albopictus* and *Ae. japonicus* (75–90%). The high values shared by the other taxa can be explained by the disparity of the morphological characters separating the processed species as well as their respective sizes.

## Data Availability

The pictures are available on request to the authors. COI sequences are available in GenBank under accession numbers MW843020 to MW843031.
